# Development of double antibody sandwich ELISA as potential diagnostic tool for rapid detection of Crimean-Congo hemorrhagic fever virus

**DOI:** 10.1038/s41598-021-93319-0

**Published:** 2021-07-19

**Authors:** Neha Shrivastava, Jyoti S. Kumar, Pragya Yadav, Anita M. Shete, Rajlaxmi Jain, Ambuj Shrivastava, Paban Kumar Dash

**Affiliations:** 1 Division of Virology, Defence Research and Development Establishment, Gwalior, 474002 India; 2 Microbial Containment Complex, National Institute of Virology, Pashan, Pune, 411 021 India

**Keywords:** Diseases, Infectious diseases, High-throughput screening, Immunological techniques

## Abstract

Crimean-Congo hemorrhagic fever (CCHF) virus, a highly pathogenic viral agent is responsible for severe fatal hemorrhagic infections in many parts of the world. The early diagnosis of CCHF infection is important for successful clinical management and epidemiological control. The nucleoprotein (NP) of CCHFV being highly conserved and immunogenic is used as early diagnostic marker. In this study, we report a rapid and sensitive double antibody based antigen capture ELISA to detect Crimean-Congo hemorrhagic fever virus (CCHFV). Highly specific polyclonal and monoclonal antibody against NP has been generated and used as capture and detector antibody respectively. The assay was able to detect viral nucleoprotein in different matrices including human serum, ticks and culture supernatant. The detection limit of the developed sandwich ELISA assay was 25 ng of purified antigen. Comparison with a real time RT-PCR revealed its detection limit to be 1000 genome equivalents of CCHFV. Further the assay was comparatively evaluated with a commercial kit employing gamma irradiated CCHFV, revealing a sensitivity and specificity of 100%. This newly developed sandwich ELISA (sELISA) with high sensitivity and specificity could be used as an efficient method for the detection of CCHF virus in humans, ticks and culture supernatant. The assay will be useful as alternate tool for diagnosis of acute infection and is amenable for screening of large scale samples in resource limited settings.

## Introduction

Crimean-Congo hemorrhagic fever (CCHF), a widespread tick borne disease is endemic in more than 30 countries in the world. The etiological agent CCHF virus belongs to the family *Nairoviridae*, genus *Orthonairovirus* and order *Bunyavirales*^1^. Since last two decades, CCHFV has spread rapidly throughout Africa, Europe and Asia and causing fatal hemorrhagic fever in naïve population. Every year, nearly 1000 cases are reported from Middle East and eastern European countries. World health organisation (WHO) has included CCHF virus in WHO R&D Blueprint as priority agent in order to opt immediate actions to prevent future epidemics. CCHFV is maintained in enzootic tick-vertebrate-tick cycle in nature, where ticks act as principal vector and reservoir^2^. Virus infected ticks, rearing in host (small and large animals) can transmit the virus to animals and humans via bite^3,4^.

CCHFV causes a fatal human infection with high mortality rate. Onset of disease coincides with sudden fever, myalgia, dizziness and bodyache leading to nausea, vomiting, diarrhoea, neural impairments, kidney and liver failure after 5th day of illness that is associated with higher mortality^5,6^. The disease also represents hemorrhagic symptoms characterized by petechial rash on mucosal surfaces, haematuria, epistaxis and gum bleeding^7,8^.

In spite of wide distribution and high mortality, vaccines for human use are not widely available. The antiviral ribavirin is non-specific and also suffers from limited efficacy. Supportive therapies including passive plasma transfusion and non-specific antivirals were attempted during initial stage of illness to reduce the mortality^9,10^. Therefore, a specific and sensitive diagnosis of CCHFV infection becomes crucial to initiate timely tracking and treatment of patients.

Currently, direct detection of CCHFV relies either on virus isolation in suitable animal model or molecular detection of virus. Both these methods are technically demanding and limited to advanced laboratory. The isolation of CCHFV involves high biohazard risk and can only be attempted in biosafety level-4 (BSL-4) laboratories^11^. Though isolation is a gold standard, however, it plays a limited role in diagnosis^12^. Although molecular assays such as real-time reverse transcriptase polymerase chain reaction (RT-PCR) can be useful for early and confirmatory diagnosis, yet most of these formats are expensive and requires high-end equipments^13^. Therefore, these formats may be good at laboratory level but difficult to implement in resource limited settings and field hospital at this point of time. Serological diagnostic kits for detection of IgM and IgG antibody are commercially available. However, by the time detectable antibody titer appears, the patient starts recovering and thus become less significant in clinical outcome of patient. Due to high mortality rate (40%), it is important to detect in early stage of infection i.e., during day 3–6 so that the supportive treatment of patient can be initiated. The antigen detection provides an easy alternative for virus detection at early stage of infection. In spite of CCHFV being a widely circulating virus, there is a lack of availability of antigen detection system around the world. Therefore, there is an urgent need to develop a safe ELISA assay for detection of CCHFV antigen in multiple matrices including serum, ticks and culture fluids. The present study is focused on development of safe, scalable and rapid sandwich ELISA for detection of CCHF virus in multiple matrices.

## Results

### Antibodies generation and titration

**Figure 1:**
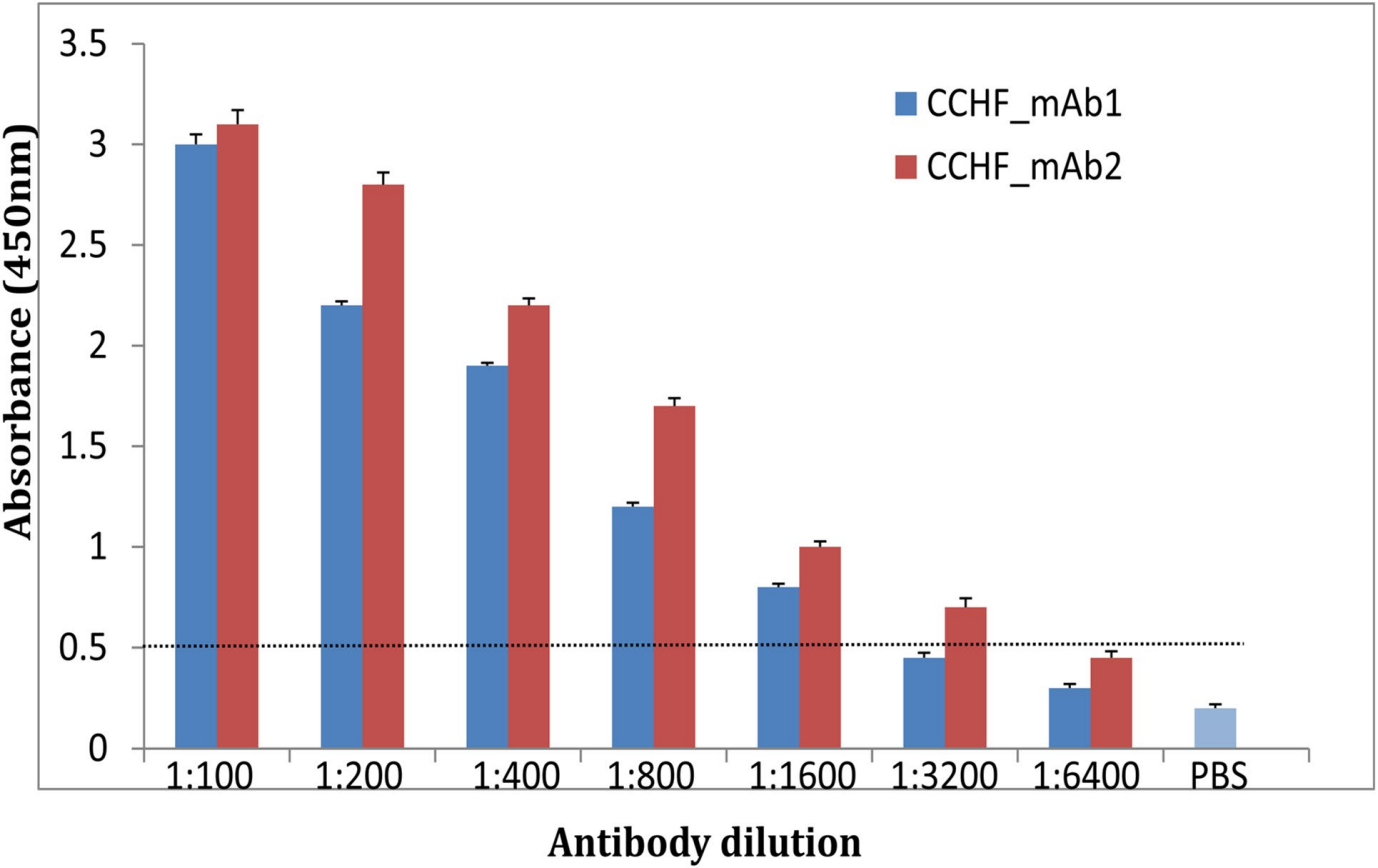
Twofold titration of monoclonal antibody (CCHF_mAb1 and CCHF_mAb2) against recombinant NP in triplicates. Monoclonal antibody 1 (CCHF_mAb1) showed reactivity titre up to 1:1600 dilution against r-NP in indirect ELISA. Monoclonal antibody 2 (CCHF_mAb2) showed the reactivity up to 1:3200 dilution against r-NP in indirect ELISA with the cut-off value of 0.5 determined by twice the OD of negative control.

The polyclonal antibody (pAb) and monoclonal antibody (mAb) against recombinant nucleoprotein were raised in rabbit and mouse respectively following the standard protocol. The titer of the generated rabbit anti-rNP polyclonal antibody was found to be 1:64,000. The concentration of purified pAb was 2.0 mg/ml. Out of ten hybridoma clones, two highly reacting hybridoma clones were further shortlisted (Supplementary Figure 1).The monoclonal antibody 2 (CCHF_mAb2) showed higher titer of 1:3200 than CCHF_mAb1 of 1:1600 (Fig. 1). The concentration of purified mAb1 and mAb2 were 1.9 mg/ml and 2.2 mg/ml respectively.

### Characterization of generatdxed antibodies and conjugation of mAb with HRP

**Figure 2:**
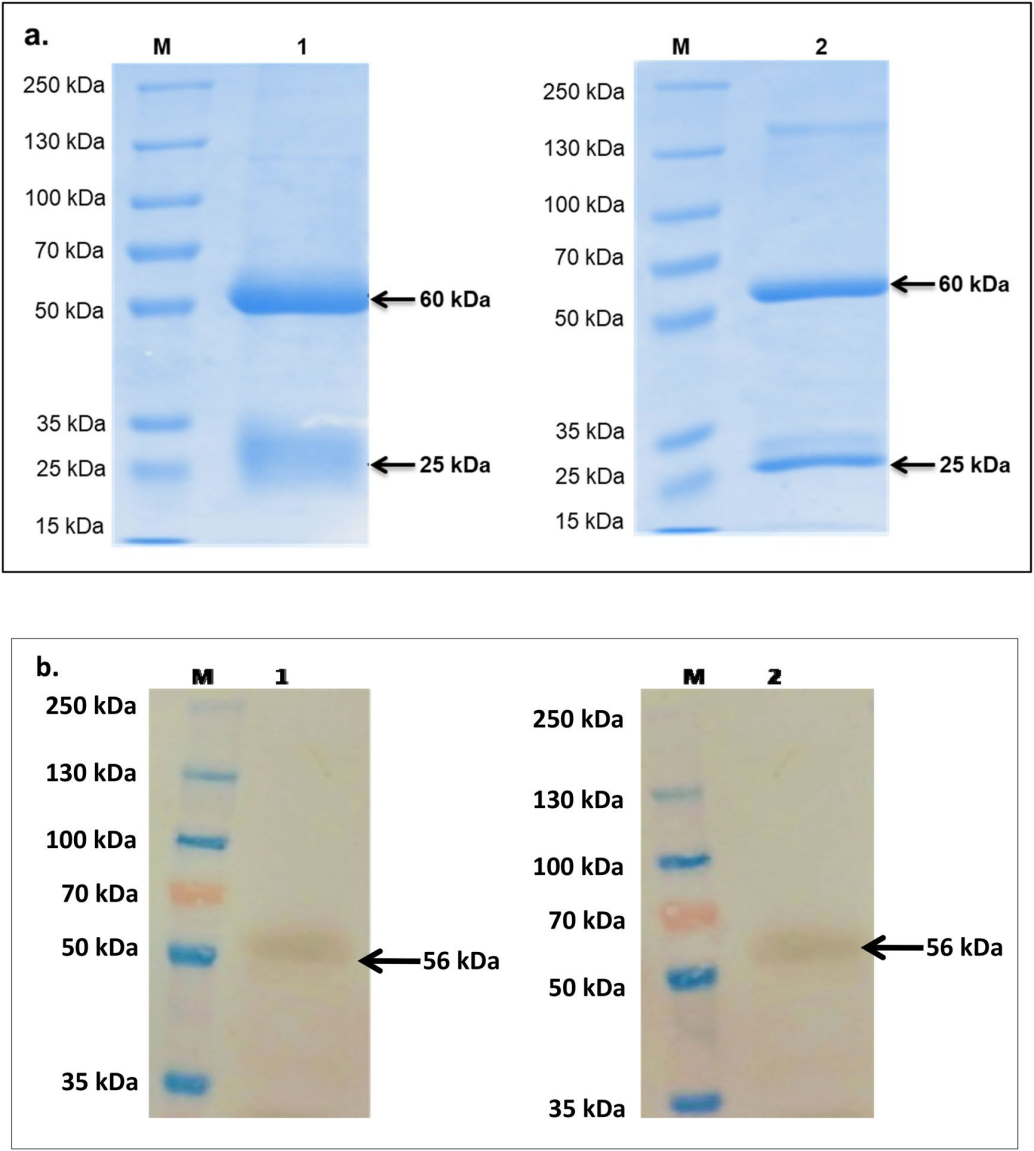
(**a**) 10% SDS-PAGE analysis of antibodiesraised against recombinant nucleoprotein. Lane 1 was loaded with polyclonal antibody and Lane 2 was loaded with monoclonal antibody (CCHF_mAb2) revealing characteristic heavy chain (60 kDa) and light chain (25 kDa) of antibodies. (**b**) Western blot analysis using polyclonal (Lane 1) and monoclonal (CCHF_mAb2) (Lane 2) antibodies showing immune reactivity with 56 kDa recombinant nucleoprotein (NP) of CCHF virus.

SDS-PAGE analysis of pAb and mAb showed the characteristic light (25 kDa) and heavy chains (60 kDa) (Fig. 2a).Western blot analysis revealed the immunoreactivity of pAb and mAb towards r-NP (56 kDa) (Fig. 2b). Following characterization, mAb_2 was successfully conjugated with HRP enzyme and the titer of the HRP conjugated mAb2 was found to be 1:1600 (Supplementary Figure 2).

**Figure 3:**
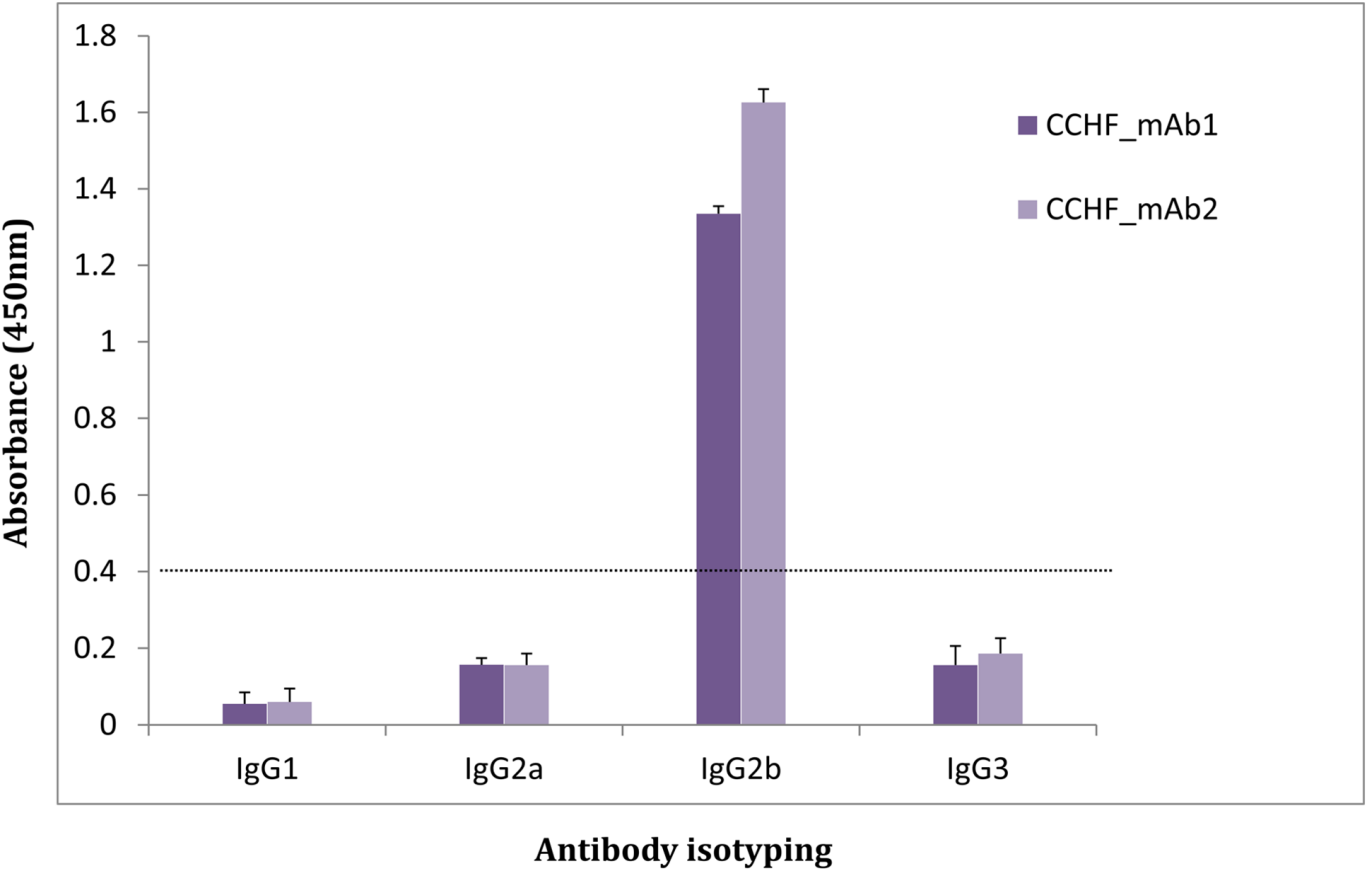
Isotyping of monoclonal antibodies (CCHF_mAb1 and CCHF_mAb2), analysed for isotypes IgG1, IgG2a, IgG2b, IgG3, using indirect ELISA in duplicates with depicted cut-off value of 0.37.

The isotyping assay revealed that both the monoclonal antibodies (CCHF_mAb1 and CCHF_mAb2) belonged to IgG2b isotype (Fig. 3). Dominance of IgG2b represents a Th1 response towards CCHF infection.

### Standardization of sandwich ELISA (sELISA)

The optimum working concentration of pAb and HRP_CCHF_mAb2 was determined with checkerboard titration. The optimum titer of pAb IgG and mAb IgG in assay was found to be 1:1000 and 1:100 respectively. The assay was optimized with one hundred and ten (n = 110) healthy human sera and twenty five (n = 25) healthy tick lysate to obtain minimal background with calculated threshold of twice the average of negative controls. The values of negative controls ranged from 0.2 to 0.37.

### Evaluation of sandwich ELISA

**Figure 4:**
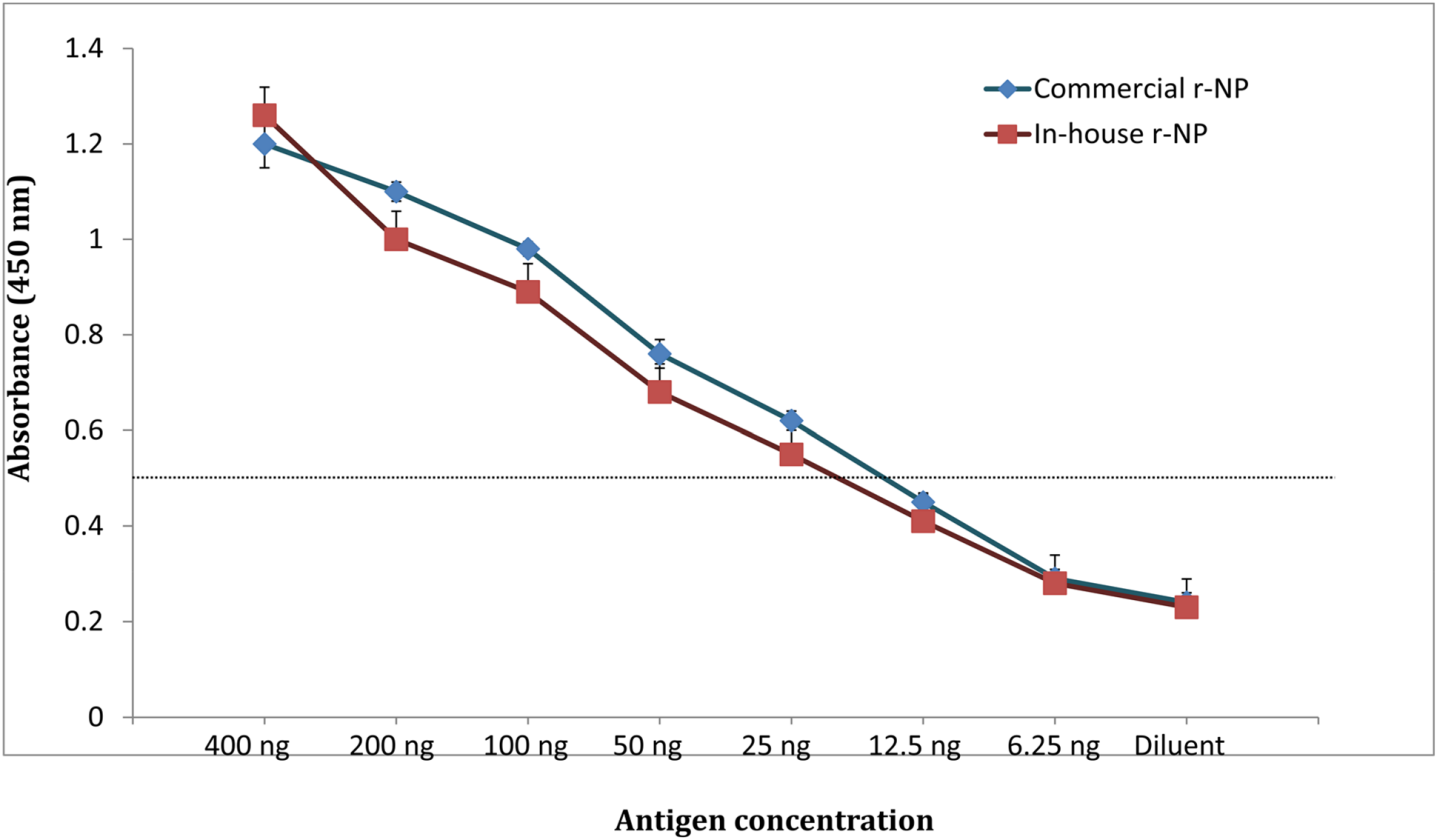
Determination of limit of detection of sandwich ELISA using in-house and commercial CCHF recombinant nucleoprotein antigen. Cut-off value was calculated as twice the value of negative control. Limit of detection was found to be 25 ng of viral nucleoprotein antigen.

Using the standardized protocol, the sELISA assay was evaluated with commercial NP antigen. The limit of detection was found to be 25 ng of viral antigen. The detection limit of the assay with in-house r-NP was also tested in parallel and similar detection limit of 25 ng was obtained (Table 1, Fig. 4). Further, on evaluation with CCHFV spiked in different matrices (culture supernatant, serum and tick lysate), sELISA was able to detect up to 10^–3^ dilution in culture supernatant and 10^–2^ dilution in both human sera and tick lysate due to matrix interference. The similar dilutions when tested in parallel with real time RT-PCR could detect up to 10^–4^ dilution. The comparison revealed a limit of detection of 1000 genome equivalents (GE) in terms of RNA copy number (Table 2).

**Table 1:** Comparative evaluation with commercial CCHF antigen and in-house recombinant antigen.

Recombinant antigen	Expression host	10^–1^ (250 ng)	10^–2^ (25 ng)	10^–3^ (2.5 ng)	10^–4^ (0.25 ng)	10^–5^ (0.025 ng)
In-house NP	*E. coli*	+	+	−	−	−
Commercial NP	Mammalian (HEK293)	+	+	−	−	−

**Table 2:** Comparative evaluation of in-house developed sELISA in different matrices with real time RT-PCR assay.

Dilution of CCHFV	CCHFV spiked in culture supernatant	CCHFV spiked in human sera	CCHFV spiked in tick lysate	CCHFV real time RT-PCR	Equivalent RNA copy number (IVT RNA)
10^–1^	+	+	+	+	10,000
10^–2^	+	+	+	+	1000
10^–3^	+	−	−	+	100
10^–4^	−	−	−	+	10
10^–5^	−	−	−	−	1

### Specificity of the CCHF sandwich ELISA assay

All the samples of DENV 1–4, KFDV, Yellow fever virus and apparently healthy individuals were found non-reactive with the developed sELISA assay (Supplementary Figure 3).

### Comparative evaluation with commercial ELISA kit

The prototype was also subjected to comparative evaluation with commercial Vecto-Crimea CHF ELISA kit (Vector Best, Russia) using γ irradiated CCHFV spiked samples. The protocol was followed as per the user manual. Data was comparatively evaluated and found that the commercial kit was able to detect up to 10^–2^ dilution of virus; while in-house prototype sELISA was able to detect the virus up to 10^–3^ in culture supernatant and 10^–2^ in both healthy human sera and tick lysate (Table 3). Sensitivity and specificity of the assay was found to be 100% with commercial antigen ELISA. The percent agreement and correlation coefficient between the two assays were 100%. However, the Vector-best antigen ELISA detected the CCHF culture fluid upto10^–2^ whereas in-house sandwich ELISA detected the dilution up to 10^–3^.

**Table 3:** Comparative evaluation of in-house developed sELISA with VectoCrimea CHF antigen ELISA kit using different dilutions of inactivated CCHFV in different matrices.

Commercial CCHF antigen ELISA kit			In-house developed sELISA		
Dilution of CCHFV	CCHFV spiked in culture supernatant	CCHFV spiked in tick lysate	CCHFV spiked in culture supernatant	CCHFV spiked in human sera	CCHFV spiked in tick lysate
10^–1^	+	+	+	+	+
10^–2^	+	+	+	+	+
10^–3^	−	−	+	−	−
10^–4^	−	−	−	−	−
10^–5^	−	−	−	−	−

## Discussion

CCHFV infection causes a fatal hemorrhagic fever in humans with high mortality rate^14^. Early detection of CCHFV becomes more crucial due to limited availability of vaccines and effective antivirals. Early diagnosis of CCHFV via RT-PCR is an expensive protocol and is not amenable in remote settings. The current disease outbreaks like a box of Pandora demands simple detection systems to track the disease and control the outbreak situations. So far, commercial serological ELISA kit (Vecto-Crimea, Russia) is available for antigen detection but is not intended for human use (mentioned in kit’s manual). Other serological in-house assays are meant to detect antibodies in different ELISA formats either in indirect ELISA or in sandwich ELISA^15–17^. It has also been reported that CCHF in cell culture does not form a clear plaque, leading to difficulties in accurate virus quantification, crucial for the development of immunodiagnostics and vaccines^18,19^.

Keeping these limitations in mind, the current study was focussed to develop a double antibody based sandwich ELISA for early detection and quantification of CCHFV in different matrices including serum sample, tick and culture supernatant. The CCHFV nucleoprotein being the most conserved structural protein among all the circulating genotypes was targeted to develop the assay. Therefore, pAb and mAb were generated against native NP to serve as capture and detector molecules respectively in sELISA format.

Following optimization, the assay was evaluated with commercial antigen produced using heterologous mammalian expression host. This NP antigen was expressed using a gene derived from CCHF strain IbAr10200, belonging to genotype III^20,21^. Though the CCHF_mAb2 was generated using in-house r-NP of a CCHFV belonging to genotype IV as an antigen^22^, its cross reaction to a heterologous genotype confirms its applicability for multiple CCHFV genotypes. The sELISA assay was able to detect both the recombinant antigens with a limit of detection of 25 ng. Sandwich ELISA on evaluation with virus, revealed the detection limit up to 1000 RNA copies which is sufficient to detect the virus in early stage. It has been reported that CCHFV RNA titers ranges from 3.2 × 10^5^ and 4.3 × 10^6^ in mild and severe human cases respectively^23^. Another study reported a viral load ≥ 1 × 10^9^ RNA copies/mL as marker for prediction of fatal outcome in CCHF infection^24^. Further, the viral load of CCHFV in tick to cause viremia in vertebrate is approximately 10^2.2^ PFU, equivalent to 1.1 × 10^5^ RNA copies^25,26^. This indicates that the assay can easily detect the virus in human and ticks in early stages of infection.

The assay also did not reveal cross reactivity with Dengue, KFD and Yellow fever virus. The comparative evaluation with commercial kit revealed a sensitivity and specificity of 100%. Furthermore, the sELISA picked an additional dilution up to 10^–3^ dilution of CCHFV in culture supernatant, indicating higher sensitivity compared to commercial kit. The sELISA detection assay reported in this study is a highly sensitive, specific and cost-effective tool for early diagnosis and surveillance of CCHFV in humans and ticks in endemic areas, particularly in resource limited settings.

## Materials and methods

### Chemicals and reagents

Recombinant nucleoprotein, Fruend’s complete and incomplete adjuvants (Sigma, USA), RPMI 1640 media and Bovine serum albumin (Thermo Fisher Scientific, USA), IgG purification kit, anti-mice HRP, anti-rabbit HRP conjugated secondary antibody (Sigma, USA), HRP-conjugation kit (Abcam, USA), Maxisorp plate (Nunc, Denmark), Mouse IgG isotyping kit and 3,3,5,5-tetramethylbenzidine (TMB) (Invitrogen, USA).

### Preparation of antigen

Recombinant nucleoprotein (NP) was used as antigen to generate specific antibodies for capturing viral antigen. A recombinant *E. coli* clone expressing CCHF NP of strain NIV112143 (GenBank Accession no. JN572089) was revived and r-NP was purified as per Shrivastava et al.^22^. Briefly, cultured and harvested cells were suspended in lysis buffer (50 mM NaH_2_PO_4_, 300 mM NaCl, and 10 mM imidazole, pH 8.0) supplemented with phenylmethylsulfonylfluoride (PMSF), lysozyme, and protease inhibiting cocktail (Sigma-Aldrich, USA). The culture was incubated at 4 °C for 30 min and sonicated at 9 pulse on/off for 30 min. The sonicated culture was centrifuged at 10,000×*g* for 20 min and the supernatant was loaded on the pre-equilibrated polypropylene column containing Ni–NTA slurry for 2 h at 4 °C for efficient binding. The column was washed with 10 bed volumes of wash buffer (50 mM NaH_2_PO_4_, 300 mM NaCl, and 20 mM imidazole, pH 8.0) and eluted with elution buffer (50 mM NaH_2_PO_4_, 300 mM NaCl, and 250 mM imidazole, pH 8.0). The protein was concentrated and dialyzed with dialysis buffer (50 mM NaH_2_PO_4_ and 300 mM NaCl) and further utilized for immunization purpose.

### Generation and purification of polyclonal antibody (pAb)

All animal experiments were approved by the Institutional Animal Ethics Committee (IAEC) constituted by the Committee for the Purpose of Control and Supervision of Experiments on Animals (CPCSEA), Ministry of Environment and Forestry, Government of India (Regd. no. 37/GO/Rbi/S/99/CPCSEA) vide protocol no. Viro-14/57/PKD. The affinity purified r-NP was used to raise polyclonal antibody in female New Zealand white rabbit. Purified NP (100 µg) along with Fruend’s complete adjuvant in 1:1 ratio (1 ml) was subcutaneously immunized in the 4 week old female rabbit, procured from DRDE Animal facility division. The booster doses were subsequently given twice within an interval of 15 days with Fruend’s incomplete adjuvant. On the day 7th of post second booster, 20 ml blood was collected from marginal ear vein of the rabbit and titrated using indirect ELISA. Immunoglobulin G was purified from antiserum with protein A sepharose column using Pure-1A antibody purification kit as per manufacturer’s instruction (Sigma, USA). Briefly, the serum was diluted with 3 volume of loading buffer and the culture supernatant was diluted with 1 volume of loading buffer. The diluted material was loaded to the column. The column was washed with 5 ml loading buffer per ml of resin. Following washing twice, the column was applied with elution buffer and the fractions were collected up to 10 ml. Assay elute fractions were analyzed for protein by absorbance at 280 nm. The positive protein fractions were pooled and neutralized with 1.5 M Tris base at pH 6.8. The protein was dialyzed in PBS (pH 7) at 4 °C for 4 h. The protein was further quantified with bicinchoninic acid (BCA) reagent and estimated at an absorbance of 562 nm. Purified IgG was titrated using indirect IgG ELISA^22^ and utilized for the development of sandwich ELISA for virus detection.

### Generation and purification of monoclonal antibody (mAb)

Monoclonal antibodies were raised against purified recombinant NP of CCHFV in in-house bred 4 weeks old female Balb/C mice at M/s Imgenex, India. On the day 7 post immunization the mice were bled from retro-orbital plexus using heparinised capillary, and the serum was separated by centrifugation and further titrated with indirect ELISA. The mouse showing high antibody titre was sacrificed by cervical dislocation and the splenocytes were fused with myeloma cells as per Kohler-Milstein’s protocol. Briefly, 7 days post second booster dose, a single cell suspension of spleenocytes was prepared aseptically in RPMI-1640 media as primary culture. A fusion cell suspension was prepared with myeloma cells Sp2/0 as per the standard protocol using HAT media. Following culture for 7–12 days, hybridoma cells were expanded further for limiting dilution to obtain monoclonal antibody secreting hybridoma clones. Ten hybridoma clones were screened and titrated using indirect IgG ELISA against NP as an antigen. The high titered monoclonal antibodies (n = 2) were purified as described above and stored at − 20 °C.

### Characterization of antibody

The purified polyclonal antibody and monoclonal antibody were electrophoresed on 10% reducing SDS-PAGE. For immunoblotting, recombinant NP was electrophoresed and subsequently transferred to nitrocellulose membrane. The membrane was blocked with 5% skimmed milk powder (SMP) in 0.1% PBST and incubated at 4 °C for overnight. The blot was washed and treated with 1:2000 and 1:500 dilution of purified polyclonal and monoclonal antibody respectively and incubated at 4 °C for 4 h. Following incubation, the membrane was washed with 0.1% PBST and treated with 1:5000 dilutions of anti-rabbit and anti-mouse HRP conjugated secondary antibody at 37 °C for 2 h. The blot was further developed with DAB/H_2_O_2_as substrate.

### Titration of monoclonal antibody

Shortlisted monoclonal antibodies were titrated with indirect ELISA assay. Briefly, 300 ng of r-NP was coated in each well of microtiter plate (Nunc, Denmark) at 4 °C for overnight. The wells were subsequently blocked with 5% SMP for 4 h, followed by washing with 0.05% PBST to remove blocking buffer. Hybridoma cell supernatants in the twofold dilution (1:100 to 1:6400) were applied to wells at 37 °C for 60 min. Following incubation, wells were washed with wash buffer (0.1% PBST) and 1:5000 dilution of anti-mouse HRP secondary antibody (Sigma, USA) was added in each well and incubated at 37 °C for 60 min. Following incubation and washing, the reaction was developed with TMB/H_2_O_2_ solution (Invitrogen, USA). The reaction was stopped with 1 N HCl and absorbance was recorded at 450 nm.

### Isotyping of monoclonal antibody

The sub-class of mAb (CCHF_mAb1 and CCHF_mAb2) were determined using mouse IgG isotyping kit (Cat. No. 88-50630-88, Invitrogen, USA). ELISA plate was coated with 100 µL/well of capture antibody in coating buffer. Each well was coated with isotype specific antibody and blocked with 250 µL of blocking buffer and incubated at room temperature for 2 h. Following incubation, 50 µL/well of assay buffer A (1×) was added to all wells and incubated at room temperature for 2 h. Wells were washed and incubated with detector antibody and incubated. Plate was washed and developed with TMB/H_2_O_2_ incubated at room temperature for 10 min and stopped with 1 N HCl. The absorbance was recorded at 450 nm wavelength.

### HRP conjugation of monoclonal antibody

The purified CCHF_mAb2 was conjugated with horseradish peroxidase enzyme (HRP) using HRP conjugation kit (Abcam, USA) as per manufacturer’s instruction to accelerate the speed compared to conventional sandwich ELISA method.

### Standardization of sandwich ELISA (sELISA)

The purified pAb and HRP_mAb2 was used as capture and detector antibodies respectively to determine the optimal concentration by checkerboard titration. In brief, 1:250, 1:500, 1:1000 and 1:1500, 1:2000 and 1:4000 dilutions of 1 mg/ml stock of polyclonal antibody were coated (100 μl/well) and incubated at 4 °C for overnight. Further the plates were blocked with 5% skimmed milk powder (SMP) in PBS and a fixed antigen concentration (100 μg/well) in healthy human serum and healthy tick lysates was added in each well for 1 h. Following incubation, the twofold serially diluted (initial concentration 1:50, 1:100, 1:500, 1:1000) HRP_mAb2 (2 mg/ml stock) was added in respective wells and incubated at 37 °C for 1 h. The plate was incubated at 37 °C for 1 h and washed, the reaction was then developed with TMB and stopped with 1 N HCl. The absorbance was recorded with ELISA plate reader (BioTek, USA).

### Evaluation of sELISA with commercial antigen

The standardized sandwich ELISA was evaluated for its efficacy in detecting a mammalian cell (HEK) expressed nucleoprotein (The Native antigen, UK). The commercial antigen (NP) was spiked in 0.05% PBST, serially tenfold diluted and tested as per standardized protocol and the limit of detection was calculated. This was also compared with in house *E. coli* expressed r-NP following the same protocol.

### Evaluation of sELISA with CCHF virus

Sandwich ELISA was evaluated with tenfold serial dilutions of gamma irradiated CCHF virus (10^–1^ to 10^–5^) in different matrices viz., culture supernatant, serum and tick lysate with standardized protocol. CCHF virus strain NIV1040505 (GenBank number MH396640-MH396642) was used in this study. For CCHFV inactivation, Co-60 source (Gamma Chamber; GC 5000) was used. For complete inactivation of the CCHFV, gamma irradiation dose of 24 kGy has been used for 2 h. Before further use, inactivation of the virus stock was confirmed by two blind passages in Vero cells. Serum and tick specimens referred to NIV during various CCHF outbreaks in India were used for the evaluation of the assay.

### Quantitative real-time PCR (qRT-PCR) of CCHFV

The same dilutions were tested with real time RT-PCR and the data was comparatively analyzed^27^. Taqman real time RT-PCR was performed using TaqMan fast virus one-step master mix in 20 µl reaction mixture (Thermo Fisher Scientific, USA) using ABI7500 Dx real time PCR system (Applied Biosystems, USA). The primer/probe set used for the real time RT-PCR targeting S-gene of CCHFV were Forward Primer: 5′-CAAAGAAACACGTGCCGCTT-3′; Reverse Primer: 5′-ATTCACCTCGATTTTGTTTTCCAT-3′ and Probe: 5′-ACGCCCACAGTGTTCTCTTGAGTGTTAGCA-3′. PCR cycling conditions included 50 °C for 30 min and at 95 °C for 2 min followed by 45 two-step cycles at 95 °C for 15 s and at 55 °C for 60 s. Following cycling, the result was analyzed from the amplification plot.

### Cross-reactivity analysis of sandwich ELISA

Cross reactivity of the sELISA assay was analysed with culture supernatants of Dengue virus serotypes [DENV-1, RR107 (KF289072); DENV-2, GWL18 (AY324614); DENV-3, ND143 (FJ644564); DENV-4, ND 73 (HM237348)], Kyasanur forest disease virus strain 12839 (MG720080) and Yellow fever virus 17D strain (KF769015).

### Statistical analysis

All the generated data was analyzed with Medcalc 2.0 software.

### Ethics statement

All methods are reported in accordance with ARRIVE guidelines. All methods were performed in accordance with the relevant guidelines and regulations. For animal experiments, the study protocol for all experiments was approved as protocol no. Viro-14/57/PKD by the Institutional Animal Ethics Committee (IAEC) constituted by the Committee for the Purpose of Control and Supervision of Experiments on Animals (CPCSEA), Ministry of Environment and Forestry, Government of India (Regd. no. 37/GO/Rbi/S/99/CPCSEA).

## Supplementary Information

Supplementary Information.
