# Supplementary material for: Development of double antibody sandwich ELISA as potential diagnostic tool for rapid detection of Crimean-Congo hemorrhagic fever virus
Source: Sci Rep. 2021 Jul 19;11:14699. doi: 10.1038/s41598-021-93319-0 (PMC8289837; doi:10.1038/s41598-021-93319-0)
Supplement: Supplementary file 1 — Supplementary Information. [file 41598_2021_93319_MOESM1_ESM.docx]

**Supplementary figures**

**S. Figure 1**

**
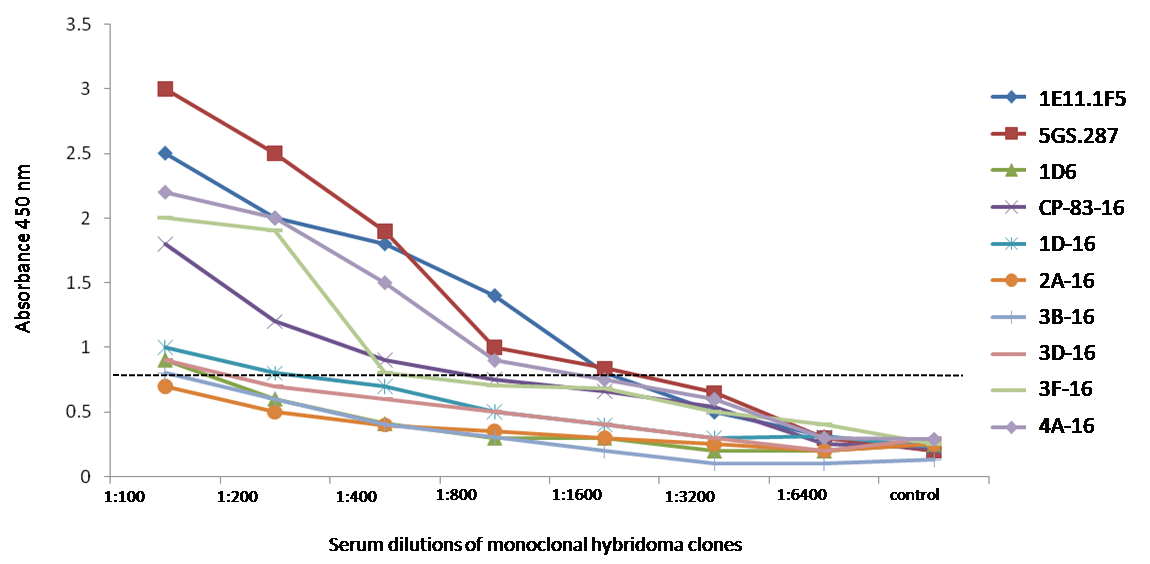
**

**Supplementary Figure 1: Titration of ten monoclonal antibody producing hybridoma clones out of which 2 highly reacting clones 1E11.1F5 and 5GS.287 were further shortlisted with revised nomenclature of CCHF_mAb1 and CCHF_mAb2 respectively**

**S. Figure 2**

**
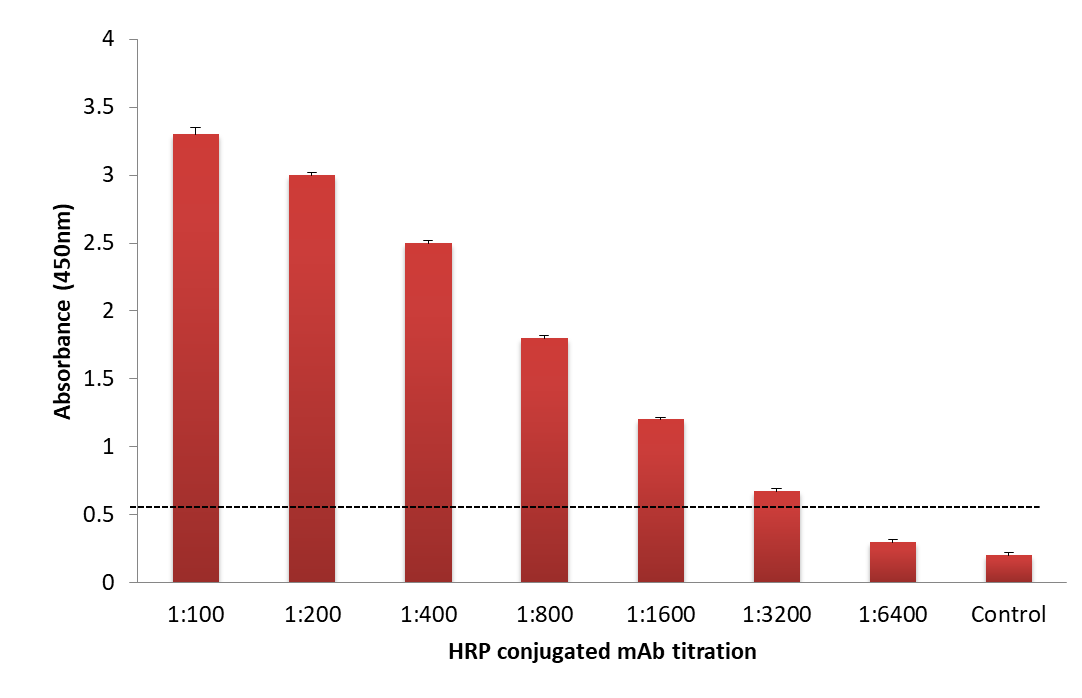
**

**Supplementary figure 2: Titration of HRP conjugated monoclonal antibody 2 against CCHF recombinant nucleoprotein using indirect ELISA with depicted cut-off of 0.5 calculated by twice the negative control**

**S. Figure 3**


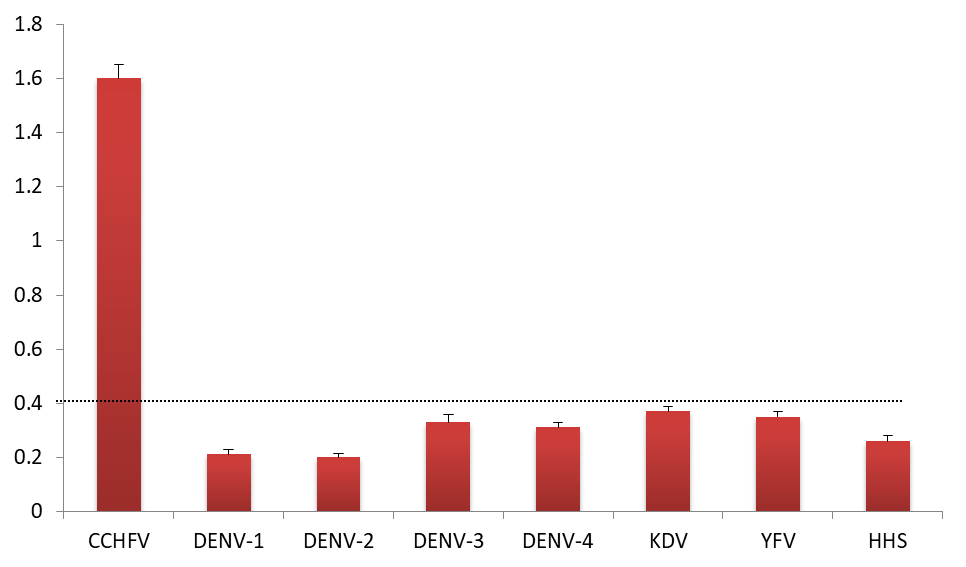


**Supplementary Figure 3: Cross reactivity analysis of sELISA with DENV 1-4, KFDV and Yellow fever and healthy human sera with depicted cut-off of 0.4 calculated by twice the value of negative control. All the DENV (1-4), KFDV and YFV culture supernatant found to be undetected by the CCHF specific sELISA assay.**
